# In Situ Formation of Silver Nanoparticles-Containing Gallic Acid-Conjugated Chitosan Hydrogels as Antimicrobial Tissue Adhesive Materials

**DOI:** 10.3390/biomimetics10110720

**Published:** 2025-10-28

**Authors:** Se-ah Kim, Da Han Hyun, Ji Hyun Ryu

**Affiliations:** 1 Department of Biomedical Materials Science, Graduate School of Jeonbuk Advanced Bio-Convergence Academy (JABA), Wonkwang University, Iksan 54538, Jeonbuk, Republic of Korea; tpdk1101@wku.ac.kr; 2 Department of Bio-Nano Chemistry, Wonkwang University, Iksan 54538, Jeonbuk, Republic of Korea; dahan93@knu.ac.kr; 3 Department of Carbon Convergence Engineering, Wonkwang University, Iksan 54538, Jeonbuk, Republic of Korea; 4 Department of Chemical Engineering, Wonkwang University, Iksan 54538, Jeonbuk, Republic of Korea; 5 Smart Convergence Materials Analysis Center, Wonkwang University, Iksan 54538, Jeonbuk, Republic of Korea

**Keywords:** gallic acid-conjugated chitosan, silver nanoparticle, metal-containing hydrogels, antimicrobial, tissue adhesive

## Abstract

Antimicrobial hydrogels have attracted considerable attention for wound treatment due to the major clinical challenges of bacterial infections, which lead to delayed tissue regeneration and chronic inflammation. In addition, the strong adhesion of antimicrobial hydrogels to tissue surfaces is essential because wounds are generally moist, topographically irregular, and continuously exposed to various biological molecules. In this study, we developed in situ formed silver nanoparticle (Ag NP)-incorporated gallic acid-conjugated chitosan (CHI-G) hydrogels as bio-inspired antimicrobial and tissue adhesive materials. Ag/CHI-G hydrogels were successfully formed by the simultaneous reduction in Ag^+^ ions with a stable dispersion of Ag NPs. No additional reduction agents or crosslinkers were required to prepare the Ag/CHI-G hydrogels. In addition, the elastic moduli of the Ag/CHI-G hydrogels increased significantly with increasing concentrations of both AgNO_3_ and CHI-G. Furthermore, the hydrogels exhibited excellent adhesion to the porcine intestinal tissue surfaces. The adhesive Ag/CHI-G hydrogels showed an inhibition of both *Escherichia coli* and *Staphylococcus aureus* with no significant cytotoxicity against NIH3T3 and CCD-18Co fibroblasts. Thus, in situ formed Ag/CHI-G hydrogels with adhesive, biocompatible, and antimicrobial properties are expected to be useful for versatile biomedical applications, such as drug delivery depots, tissue engineering hydrogels, and wound dressing materials.

## 1. Introduction

Antimicrobial hydrogels are preferred biomaterials for preventing pathogenic microbial infections caused by bacteria and fungi during treatment of wounds. In general, infection at wound sites is a major clinical concern because it delays the healing process by disrupting epithelial regeneration and tissue remodeling and may eventually cause prolonged local inflammation, tissue necrosis, secondary infections, and progression into chronic wounds [1,2,3]. Chronic wounds affect 2.4–4.5 million individuals annually in the United States, with an average duration of 12–13 months and a recurrence rate reaching up to 70% [4]. Approximately 78% of these chronic wounds are associated with biofilm formation, which serves as a critical factor in facilitating bacterial communication and enhancing resistance to antibiotics [5,6,7]. Therefore, it is highly desirable to develop biocompatible antimicrobial hydrogels for wound treatment.

A significant issue in applying antimicrobial hydrogels for wound treatment is tissue adhesiveness. For the application of antimicrobial biomaterials, effective pathogen eradication and durable adhesion to the wound site are essential for achieving therapeutic efficacy within the complex wound microenvironment [8,9]. Detachments or displacements of dressing materials often occur because wound surfaces are typically moist, topographically irregular, and continuously exposed to various biological exudates (i.e., enzymes, proteins, and fluids), which frequently interfere with their effective adherence to the wound site [10,11]. Phenolic polymer-based bioadhesive materials have attracted significant scientific interest because of their distinct chemical functionalities and versatile tissue interactions [12,13,14,15]. For instance, catechol-functionalized hyaluronic acid/tannic acid/dopamine-modified carbon particle composite hydrogels demonstrated rapid gelation within seconds via oxidative crosslinking, displaying enhanced adhesive strength, and facilitated accelerated wound healing in full-thickness skin wound models [14]. Catechol-conjugated chitosan provides the dual functionality of bioadhesion and controlled drug release, eliciting tissue regeneration and cellular recovery in acute skin wound rat models [15]. Thus, phenolic compound-conjugated polymer-based hydrogels are promising candidates for wound management because of their diverse interactions with biological tissues and ability to maintain strong adhesion under moist conditions.

Chitosan, a naturally occurring polysaccharide, is used to treat wounds because of its biocompatibility and biodegradability and induces antimicrobial effect via electrostatic interactions between its cationic amino groups and negatively charged bacterial or cellular membranes [16,17]. To enhance its therapeutic performance, phenolic compound-conjugated chitosan (e.g., catechol- or gallol-containing chitosan) inspired by nature have been developed [18,19,20]. Polyphenols are abundantly found in the plant kingdom that are known to have strong adhesive properties via physical and chemical interactions. By conjugation of phenolic compounds into the chitosan backbones, the chitosan derivatives show enhanced tissue adhesiveness with versatile functionality [21,22]. For instance, gallic acid-conjugated chitosan (CHI-G) not only retains its intrinsic properties but also introduces additional adhesive and antioxidative properties useful for versatile biomedical applications [19,20]. CHI-G-based hydrogels exhibited excellent tissue adhesive properties (detachment stress of 47 kPa), which were far higher than those of chitosan (4 kPa) [23]. Furthermore, CHI-G hydrogels incorporating nano- or micro-particles have demonstrated multifunctional properties, including antimicrobial activity, reactive oxygen species (ROS) scavenging efficacy, and tissue adhesive properties, positioning them as advanced wound dressing platforms with promising utility for therapeutic wound healing [24,25].

Silver nanoparticles (Ag NPs) are standard antimicrobial nanomaterials owing to their broad-spectrum antimicrobial activity against Gram-positive and Gram-negative bacteria and fungi [26,27]. More importantly, Ag NPs have been reported to be effective against antibiotic-resistant strains [28,29,30,31]. The multifaceted antimicrobial mechanism of Ag NPs involves the disruption of microbial membranes, release of Ag^+^ ions causing protein denaturation and DNA damage, and induction of oxidative stress through ROS generation [32,33,34,35,36,37]. However, Ag NPs are associated with potential risks and toxic effects [38,39]. Strong reducing agents (e.g., sodium borohydride) are generally required to prepare Ag NPs, which can generate toxic byproducts and increase process complexity [40,41,42,43,44]. Furthermore, Ag NPs tend to aggregate in aqueous environments due to their high surface energy, which potentially diminishes their antimicrobial efficacy by reducing their available surface area [45]. To address cytotoxicity issues, Ag NPs are frequently embedded within biocompatible polymer matrices after the removal of additives, which improves their biocompatibility without squandering their antimicrobial properties [46,47,48,49].

**Figure 1:**
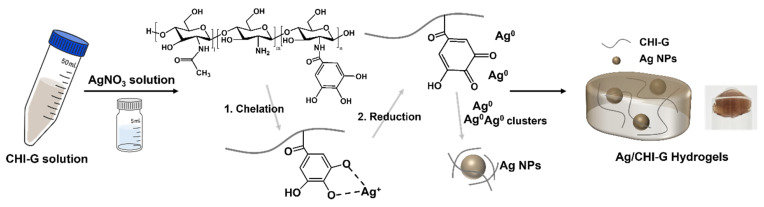
Schematic representative of preparation and formation of Ag/CHI-G hydrogels.

We hypothesized that the in situ formation of Ag NPs without additives in biocompatible phenolic compound-conjugated polymeric hydrogels could reduce cytotoxicity and improve adhesive properties. In this study, we developed in situ formed Ag NP-containing CHI-G (Ag/CHI-G) hydrogels with biocompatible, tissue adhesive, and antimicrobial properties (Figure 1). As previously reported, CHI-G shows an intrinsic capability to spontaneously reduce the Ag^+^ ions to metallic Ag^0^ autonomously without the addition of reductants [48,49], and CHI-G-based hydrogels without Ag NPs exhibit strong adhesion to biological tissues and hydrated surfaces [50]. The addition of AgNO_3_ to the CHI-G solution promoted the in situ formation of Ag NPs via the reduction capability of CHI-G, with the formation of three-dimensional structures of Ag/CHI-G without external reducing agents. The Ag/CHI-G hydrogels showed increased elastic moduli compared with those of CHI-G alone. In addition, the hydrogels exhibited enhanced tissue adhesion without inducing cytotoxicity. Furthermore, a significant inhibition of both *Escherichia coli* and *Staphylococcus aureus* by the hydrogels was observed, indicative of the excellent antimicrobial properties of Ag/CHI-G hydrogels. In summary, Ag/CHI-G hydrogels prepared by the addition of AgNO_3_ into CHI-G solutions showed enhanced tissue adhesion and antimicrobial properties without significant cytotoxicity. Thus, the in situ formed Ag/CHI-G hydrogels are expected to be utilized as biocompatible, antimicrobial, and tissue adhesive materials for versatile biomedical applications.

## 2. Materials and Methods

### 2.1. Materials

Chitosan (MW: 190–310 kDa) and hydrochloric acid (HCl) were obtained from Sigma-Aldrich (St. Louis, MO, USA). Sodium hydroxide (NaOH) was purchased from Kanto Chemicals. Co. Inc. (Tokyo, Japan). Gallic acid, 1-Ethyl-3-(3-dimethylaminopropyl)carbodiimide (EDC), and N-Hydroxysuccinimide (NHS) were purchased from TCI (Tokyo, Japan). Silver nitrate (AgNO_3_) was purchased from Duksan Pure Chemicals (Ansan, Republic of Korea).

### 2.2. Synthesis of Gallic Acid-Conjugated Chitosan

Gallic acid-conjugated chitosan (CHI-G) was synthesized via standard carbodiimide chemistry, according to previous research [22,51,52]. Briefly, chitosan (1 g) was dissolved in 1 N HCl (2 mL), followed by the addition of distilled and deionized water (DDW) to a final volume of 80 mL. The pH of the solution was adjusted to 5.0 using 1 N NaOH. Gallic acid (1 g), EDC (1.2 g), and NHS (0.7 g) were dissolved in a solvent mixture of deionized water and ethanol (3:1, *v*/*v*) and added to the chitosan solution. The reaction mixture was stirred at room temperature for 12 h, maintaining a pH of 5.0 throughout the reaction. After the reaction, the resulting solution was dialyzed against pH 2.0 aqueous solution for 3 d using a dialysis membrane (MWCO: 12–14 kD), followed by an additional 4 h of dialysis against deionized water.

### 2.3. Characterizations of CHI-G

The synthesis of CHI-G was confirmed using ^1^H Nuclear Magnetic Resonance (NMR) and Ultraviolet-Visible (UV-Vis) spectroscopy according to previous report [22]. The ^1^H NMR spectrum of CHI-G was obtained using ^1^H NMR spectroscopy (Bruker Avance III, 500 MHz; Billerica, MA, USA). Deuterium oxide (D_2_O) was used as the solvent. The degree of gallic acid substitution was calculated by comparing the integrated areas of a gallol group (2H) and an acetyl group (3H) on a CHI-G. To further confirm the conjugation of gallic acid to the chitosan backbone, the UV-Vis spectra of CHI-G were obtained using a UV-Vis spectrophotometer (UV-2450, Z-202201147260; Shimadzu, Kyoto, Japan) at the Core Facility of Wonkwang University, supported by NFEC. CHI-G was dissolved in DDW at a concentration of 0.025 mg/mL and GA solutions were prepared as a function of concentration (0.001, 0.005, 0.01, 0.025, 0.05, and 0.1 mg/mL). The degree of GA substitution was calculated by comparing the absorbance of CHI-G at 265 nm with that of the GA standard curves. The degree of GA substitution in the chitosan backbone was 7.6% (^1^H NMR) and 6.6% (UV-Vis spectroscopy). The difference in the degree of GA substitution of CHI-G between two different measurements might be due to the potential error of a deacetylation rate provided by a manufacturer.

### 2.4. Study of Formation of Ag NPs Using CHI-G

To confirm the in situ formation of Ag NPs, UV-Vis spectroscopic studies of mixed solutions of CHI-G and AgNO_3_ were performed. The UV-Vis spectra of CHI-G with AgNO_3_ were monitored after 0 h and 3 d. In addition, the characteristic surface plasmon resonance (SPR) peak of Ag NPs at approximately 420 nm was monitored. The concentrations of CHI-G and AgNO_3_ were 0.1 wt% and 7 mM, respectively. The CHI-G and AgNO_3_ solutions were used as controls. All measurements were performed in triplicates.

### 2.5. Particle Size Distributions of Ag NPs Using CHI-G

The particle size distribution of the Ag NPs prepared using CHI-G was measured by dynamic light scattering (DLS; ELSZneo, Otsuka Electronics, Osaka, Japan). Disposable polystyrene cuvettes were used for all samples, and the scattering angle was 90° at 25 °C. In addition, morphological analysis of the Ag NPs prepared using CHI-G was performed using scanning electron microscopy (SEM; Hitachi S-4800, Z-202201147255; Hitachi, Ltd., Tokyo, Japan) at the Core Facility of Wonkwang University, supported by NFEC. The obtained SEM images were further analyzed using the ImageJ software (Version 1.54g, National Institutes of Health, Bethesda, MD, USA), and the results were compared with those obtained by DLS to confirm the formation and characteristics of Ag NPs. The concentrations of CHI-G and AgNO_3_ were 0.1 wt% and 7 mM, respectively. All measurements were performed in triplicates.

### 2.6. Preparation of Ag/CHI-G Hydrogels

The Ag/CHI-G hydrogels were prepared by adding AgNO_3_ solution was added to a CHI-G solution. Briefly, CHI-G was dissolved in DDW, the pH of the CHI-G solution was adjusted to 6.0–6.5, and AgNO_3_ solution was then added. The mixture was incubated at room temperature for over three days to promote in situ formation of Ag NPs within the CHI-G hydrogel network. CHI-G solutions were prepared at following concentrations: 2, 3, 4, 6, and 8 wt%, and the final concentration of CHI-G was fixed at 4 wt%. In addition, the concentration of the AgNO_3_ solution varied from 5 to 11 mM considering previous reports of antimicrobial activities and cytotoxicity [53,54].

### 2.7. Morphological Analysis of Ag/CHI-G Hydrogels

Morphological analysis of the Ag/CHI-G hydrogels was performed by SEM. Ag/CHI-G hydrogels were obtained by reacting 4 wt% CHI-G with aqueous AgNO_3_ (5, 7, or 11 mM), followed by freeze-drying. All specimens were sputter-coated with platinum prior to the SEM analysis.

### 2.8. Rheological Analysis of Ag/CHI-G Hydrogels

The rheological properties of the Ag/CHI-G hydrogels were evaluated using a rotational rheometer equipped with a 20 mm parallel plate geometry. The elastic (G′) and viscous (G″) moduli were determined using frequency sweep measurements conducted at a gap of 0.15 mm, a temperature of 25 °C, and a frequency range of 0.1–10 Hz. Ag/CHI-G hydrogels were prepared by dissolving 4 wt% CHI-G in distilled water, followed by the addition of AgNO_3_ (5, 7, or 11 mM). For the control group, CHI-G was prepared in distilled water at concentrations of 2, 3, 4, 6, and 8 wt%. All measurements were performed in triplicates.

### 2.9. Swelling Ratios and Relative Remaining Weights of Ag/CHI-G Hydrogels

The swelling ratios of the Ag/CHI-G hydrogels were determined in PBS (pH 7.4). Ag/CHI-G hydrogels were prepared by reacting 4 wt% CHI-G with aqueous AgNO_3_ solutions at concentrations of 5, 7, or 11 mM, followed by freeze-drying. All samples were immersed in PBS (0.5 mL) and incubated at 37 °C for 24 h. Excess surface water on the swollen samples was carefully removed by gentle blotting with filter paper for 3 s. CHI-G without additives was used as a control. The swelling ratio was calculated as follows:

Swelling ratio (%) = (W_wet_ − W_dry_)/W_dry_ × 100 (%), (1)

where W_wet_ is the weight of the swollen sample and W_dry_ is the initial dry weight.

The relative remaining weights of the Ag/CHI-G hydrogels were analyzed to evaluate their in vitro stability. Ag/CHI-G hydrogels were prepared by reacting 4 wt% CHI-G with aqueous solutions of 5, 7, or 11 mM AgNO_3_, followed by freeze-drying. All samples were immersed in 0.5 mL PBS (pH 7.4) and incubated at 37 °C. At predetermined time intervals (30 min, 3 h, 12 h, 1 d, 3 d, 5 d, and 7 d), the samples were retrieved, and excess surface water on the wet samples was carefully removed by gentle blotting with filter paper for 3 s. The samples were then rinsed by dipping in distilled water at 37 °C for 5 s, excess surface moisture was carefully removed using the previously described method, and the samples were subsequently freeze-dried. CHI-G without additives was used as a control. Relative remaining weight was calculated using the following equation:

Remaining weight (%) = W_t_/W_dry_ × 100, (2)

where W_t_ represents the dry weight at the designated time, and W_dry_ represents the initial dry weight.

### 2.10. Tissue Adhesive Properties of Ag/CHI-G Hydrogels

The tissue adhesive properties of the Ag/CHI-G hydrogels were evaluated using a universal testing machine (UTM; Instron 5583, Instron, Norwood, MA, USA) with a 50 N load cell. Porcine intestinal tissues were used to measure tissue adhesiveness in consideration of wound treatment. In addition, fresh and hydrated tissues were used for tissue adhesiveness measurements without the control of humidity and temperatures. For the tensile strength tests, the PET films were cut into pieces measuring 1 × 5 cm^2^. In addition, fresh porcine intestinal tissues were trimmed into 1 × 1 cm^2^ sections and affixed to the ends of the prepared PET films using commercially available cyanoacrylate adhesives that were applied only to the outer edges of the bonding area. After washing and removal of excess moisture, Ag/CHI-G hydrogels (0.2 mL) were applied to the tissue surface, and another PET film with the attached tissue was placed in contact to allow adhesion of the hydrogels between the two tissue sections. Ag/CHI-G hydrogels were prepared using 4 wt% CHI-G with aqueous AgNO_3_ solutions (5, 7, or 11 mM), and for comparison, 4 wt% CHI-G without AgNO_3_ was used. For the control group, no polymers were applied between the tissues. Tissue adhesion data were collected by pulling the UTM probe with a loading rate of 1 mm/min. All measurements were performed in triplicate.

### 2.11. Cell Viability Assay of Ag/CHI-G Hydrogels

To evaluate the cytotoxicity of the Ag/CHI-G hydrogels, cell viability tests were performed using hydrogel extracts. Briefly, Ag/CHI-G hydrogels were prepared by mixing CHI-G (4 wt%) and AgNO_3_ solutions (5, 7, or 11 mM). The hydrogels were applied onto 96-well plates (50 µL/well), cured for 12 h, and subsequently incubated with culture media of Dulbecco’s Modified Eagle Medium (DMEM; Welgene, Gyeongsan, Republic of Korea) supplemented with 10% heat-inactivated bovine calf serum (BCS) and 1% antibiotic-antimycotic (A.A.; Gibco, Thermo Fisher Scientific, Waltham, MA, USA). After the hydrogels were incubated at 37 °C in a humidified 5% CO_2_ atmosphere for 24 h, the extracts from the hydrogels were obtained. NIH/3T3 mouse fibroblasts and CCD-18Co human colon fibroblasts were used for cytocompatibility testing because fibroblasts play a significant role in the wound healing process. For cell viability quantification, 200 µL of cell suspension (1 × 10^4^ cells/well) was seeded into 96-well plates. After removing the media, the hydrogel extracts (200 µL) were added to the well plates. After another 24 h of incubation, MTT solution was added to achieve a final concentration of 0.5 mg/mL, followed by incubation at 37 °C for 2 h. The resulting formazan crystals were dissolved in 100 µL DMSO, and absorbance was measured at 570 nm using a microplate reader (iMark; Bio-Rad, Hercules, CA, USA).

### 2.12. Live/Dead Assay on Ag/CHI-G Hydrogel-Coated Surfaces

To further monitor cytotoxicity, a live/dead assay was performed on the Ag/CHI-G hydrogel films on the culture dish. Briefly, the Ag/CHI-G hydrogels were applied onto cover glasses placed in 4-well cell culture plates (80 µL/well). The hydrogels were air-dried at room temperature for approximately 12 h to create thin films, which were then rinsed three times with PBS. Cell suspensions of NIH/3T3 were seeded onto the Ag/CHI-G-coated cover glasses in 4-well plates at a final volume of 500 µL/well and incubated for 24 h. Cell viability was assessed using the LIVE/DEAD Cell Imaging Kit (Calcein-AM/BOBO-3 Iodide, Cat#R37601; Thermo Fisher Scientific, Waltham, MA, USA), and cells were stained for 15 min at room temperature before imaging. Phase-contrast and fluorescence images were acquired using inverted microscopy and confocal laser scanning microscopy, respectively.

### 2.13. Zone of Inhibition Assay of Ag/CHI-G Hydrogels

The antibacterial activities of the Ag/CHI-G hydrogels were evaluated using the standard disk diffusion method. Briefly, the Ag/CHI-G hydrogels were applied to sterile paper disks (diameter: 10 mm) and placed on LB agar plates inoculated with *E. coli* (Gram-negative, KCTC 1682) or *S. aureus* (Gram-positive, KCTC 3881). PBS-treated disks were used as negative controls. After incubation at 37 °C for 24 h, the diameters of the zones of inhibition (ZOIs, mm) surrounding the disks were measured. All experiments were performed in triplicates.

### 2.14. Evaluation of Antibacterial Activity in Liquid Culture

To further monitor the antibacterial activity of Ag/CHI-G composites, liquid cultures of *E. coli* and *S. aureus* were performed. Ag/CHI-G hydrogels (50 mg) on sterile paper disks were added to 5 mL of LB medium containing 2 × 10^6^ cells/mL and incubated at 37 °C while shaking at 250 rpm in a shaking incubator. After a 12 h incubation, the supernatants were collected and transferred to a 96-well plate (200 µL/well), and OD_595_ was recorded using a microplate reader. All experiments were performed in triplicates.

### 2.15. Statistical Analysis

Statistical analysis was performed using one-way analysis of variance (ANOVA) followed by Tukey’s post hoc test for multiple comparisons using GraphPad Prism 9 (GraphPad Software, San Diego, CA, USA). The significance level was set at * *p* < 0.01 compared with the control group.

## 3. Results and Discussion

### 3.1. Synthesis and Characterizations of CHI-G

**Figure 2:**
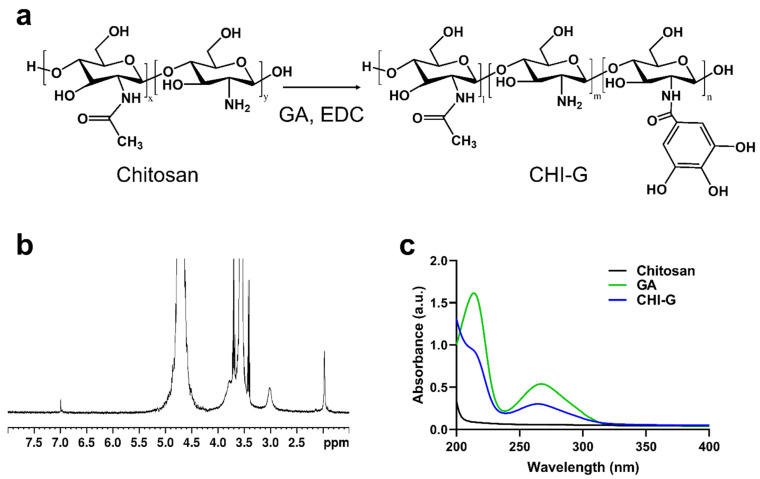
(**a**) Synthesis and chemical structures of CHI-G. (**b**) ^1^H NMR and (**c**) UV-Vis spectra of CHI-G.

As shown in Figure 2a, gallic acid-conjugated chitosan (CHI-G) was synthesized using carbodiimide chemistry, enabling the formation of amide bonds between the primary amine groups of chitosan and carboxylic acid groups of gallic acid (GA). The conjugation of GA to the chitosan backbone was confirmed using ^1^H NMR and UV-Vis spectroscopy. As shown in Figure 2b, a peak at 6.8–7.2 ppm in the ^1^H NMR spectrum of CHI-G was caused by aromatic protons of the gallol groups indicating the conjugation of gallol groups in the chitosan backbones. As previously reported, the peak around 6.9 ppm in the ^1^H NMR spectrum of CHI-G can be assigned to the gallol protons [55]. In addition, a characteristic absorbance peak at 265 nm, caused by the gallol moieties, was observed in the UV-Vis spectra of CHI-G (Figure 2c). The absorption at 260–270 nm in the UV-Vis spectra confirmed the conjugation of gallol into the polymeric backbones [56].

### 3.2. Synthesis and Characterizations of Ag NPs Using CHI-G

**Figure 3:**
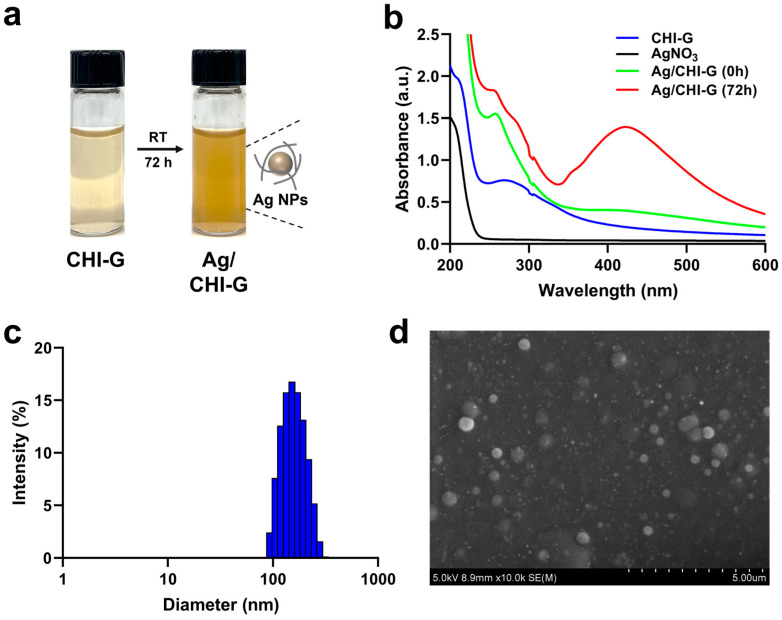
(**a**) Photographic images of CHI-G and Ag/CHI-G solutions. (**b**) UV-Vis spectra of CHI-G, AgNO_3_, and Ag/CHI-G solution as a function of time (0 and 72 h). (**c**) Particle size distribution of Ag/CHI-G complexes (0.1 wt% CHI-G and 7 mM AgNO_3_) after 72 h incubation. (**d**) SEM image of Ag/CHI-G complexes after 72 h incubation.

The Ag NPs were synthesized based on the reduction capability of the pendent gallol groups in CHI-G. As shown in Figure 3a, a color change from yellow to brown was observed before and after the addition of AgNO_3_ to the CHI-G solutions. In general, gallic acid, which bears three phenolic hydroxyl groups, functions as both a reducing agent and a chelating ligand for metal ions [57]. As reported previously, the gallol groups of CHI-G undergo deprotonation and form phenoxide ions that simultaneously chelate Ag^+^ ions, thereby stabilizing them within the polymer matrix and promoting controlled nanoparticle growth [48]. Following chelation, the gallol groups donate electrons to Ag^+^ and reduce it to metallic Ag (Ag^0^), leading to the formation of Ag^0^ clusters and aggregation, resulting in the generation of Ag NPs embedded within the CHI-G hydrogel network [48]. To confirm the successful formation of Ag NPs, the UV-Vis spectra of solutions of CHI-G and AgNO_3_ mixture were measured (Figure 3b). Immediately after mixing CHI-G and AgNO_3_, the mixtures exhibited no significant absorbance peak around 420 nm. However, after three days of reaction, the mixture showed a distinct absorbance peak near 420 nm. This peak is characteristic of surface plasmon resonance (SPR) and indicates the formation of Ag NPs. Evaluating the size of the nanoparticles, dynamic light scattering (DLS) analysis revealed an average particle size of approximately 159.96 ± 45.85 nm (Figure 3c). Additionally, the SEM images of the mixture after three days supported the particle distributions of the Ag NPs (Figure 3d). The silver nanoparticles exhibited a spherical shape with an average size of 238.26 ± 78.38 nm, confirming that well-dispersed Ag NPs were successfully generated. The discrepancy in the average particle sizes obtained from DLS and SEM analyses might be caused by methodologies of the two techniques. While DLS determines the hydrodynamic diameter of particles in their solution state, SEM provides measurements under dehydrated and vacuum conditions, where particle aggregation during the drying process may additionally influence the observed size [58]. Particularly, SEM images of Ag/polymer composite NPs show slightly different sizes due to the capillary forces and coating effects on the substrates during the dehydration steps [59].

### 3.3. Preparation and Characterization of Ag NPs-Containing CHI-G Hydrogels

**Figure 4:**
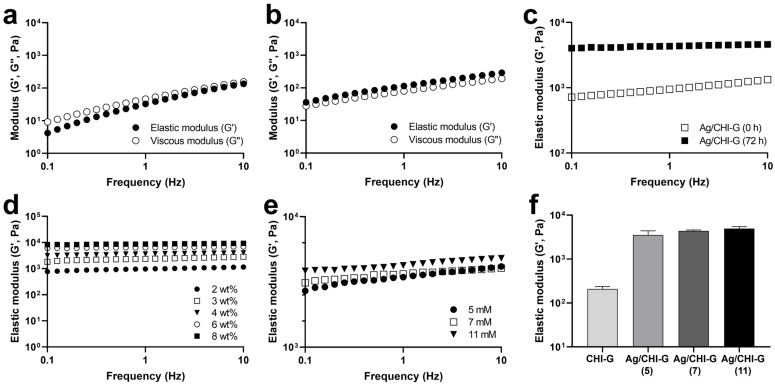
(**a**,**b**) Frequency sweep measurements of CHI-G at concentrations of (**a**) 2 wt% and (**b**) 4 wt%. (**c**) Elastic modulus of Ag/CHI-G hydrogels changes right after addition of AgNO_3_ and subsequent 72 h incubations. (**d**,**e**) Effects of (**d**) CHI-G (2–8 wt%) and (**e**) AgNO_3_ concentrations (5–11 mM) on G′ values. (**f**) Average G′ values of CHI-G and Ag/CHI-G hydrogels (5–11 mM) after 72 h incubations.

The Ag/CHI-G hydrogels were spontaneously formed by the formation of Ag NPs in the concentrated CHI-G networks when AgNO_3_ solution was added to the CHI-G hydrogels. To prepare the Ag/CHI-G hydrogels, AgNO_3_ solutions (5, 7, or 11 mM) were added to CHI-G, as illustrated in Figure 1. The rheological analysis of Ag/CHI-G hydrogels was performed to monitor the G′ and G″ values according to the concentrations of CHI-G and AgNO_3_. The CHI-G above 4 wt% concentrations showed high G′ values (114.6 Pa) compared to G″ values (80.0 Pa) at a frequency of 1 Hz (Figure 4b), whereas no gelation was observed in 2 wt% CHI-G (Figure 4a). As previously reported, CHI-G alone forms three-dimensional networks via hydrogen bonds at an early stage [60]. The increase in the G′ values (1.01 ± 0.16 kPa) was observed for Ag/CHI-G hydrogels with AgNO_3_ (7 mM) (Figure 4c). In addition, the G′ values of Ag/CHI-G hydrogels further increased to 4.38 ± 0.23 kPa after 72 h incubations (Figure 4c). Figure 4d presents the effects of CHI-G concentrations in G′ values of Ag/CHI-G hydrogels with AgNO_3_ (7 mM) after 72 h incubations, which indicate that the G′ values were controlled by the changes in CHI-G concentrations. In addition, the concentration of AgNO_3_ affected the G′ values of Ag/CHI-G hydrogels with 4 wt% CHI-G (Figure 4e). The G′ values of Ag/CHI-G hydrogels increased to 3.54 ± 0.85 kPa with 5 mM AgNO_3_, 4.38 ± 0.23 kPa with 7 mM AgNO_3_, and 4.94 ± 0.53 kPa with 11 mM AgNO_3_ (Figure 4f). This suggests that the generation of Ag NPs could serve as an effective strategy for enhancing the G′ of hydrogels.

**Figure 5:**
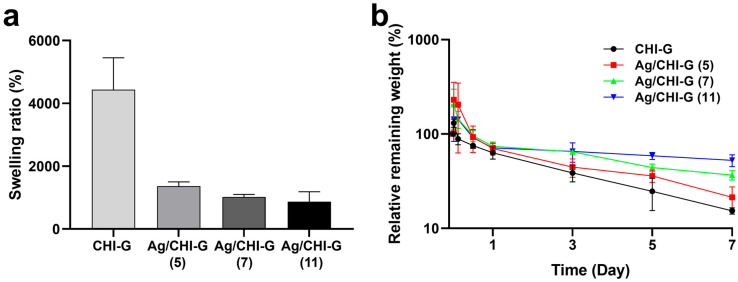
(**a**) Swelling ratios of Ag/CHI-G hydrogels as a function of AgNO_3_ concentrations. (**b**) Relative remaining weights of Ag/CHI-G hydrogels in pH 7.4 PBS at a predetermined time interval.

The swelling ratios and in vitro stabilities of the Ag/CHI-G hydrogels were measured. As shown in Figure 5a, the equilibrium swelling ratios of the Ag/CHI-G hydrogels were 1386.0 ± 132.4%, 1021.3 ± 83.3%, and 872.7 ± 314.2% with 5, 7, and 11 mM AgNO_3_ solutions, respectively; these were far lower than that of CHI-G hydrogels alone (4435.3 ± 1016.5%). As previously reported, the addition of metal NPs, including Ag NPs, can decrease the swelling ratio of hydrogels [61]. In addition, in vitro stability tests of the CHI-G and Ag/CHI-G hydrogels with different AgNO_3_ concentrations (5, 7, and 11 mM) were performed (Figure 5b). The relative remaining weight of all hydrogels was ~70% of their initial weight after 1 d, which might be due to dissociation of un-crosslinked fraction of hydrogels. However, the Ag/CHI-G hydrogels showed enhanced in vitro stability compared to the CHI-G hydrogels alone. After 7 d, the relative remaining weight of Ag/CHI-G hydrogels with 11 mM AgNO_3_ was 52.7 ± 7.6% of its original weight, which is far higher than those of CHI-G hydrogels (15.3 ± 1.2%) and Ag/CHI-G hydrogels with 5 mM (21.3 ± 6.1%) and 7 mM AgNO_3_ (36.7 ± 4.2%). This indicates that the presence of Ag NPs in the CHI-G networks enhanced the in vitro stability of Ag/CHI-G hydrogels.

**Figure 6:**
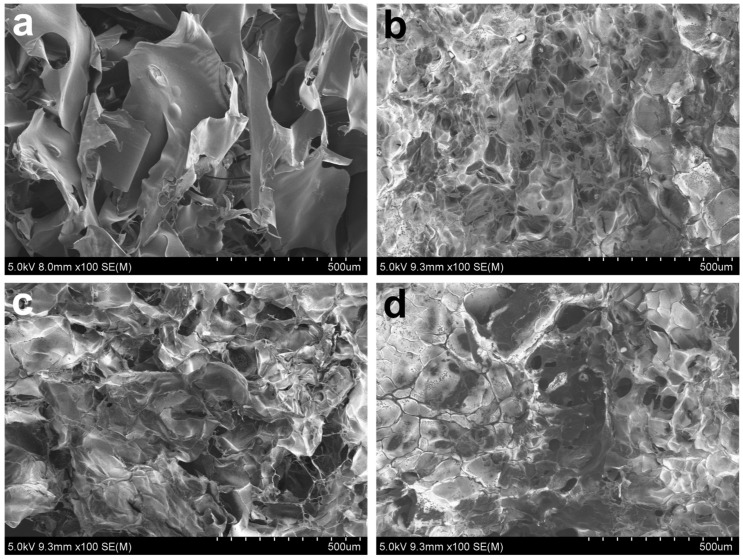
SEM images of (**a**) CHI-G and (**b**–**d**) Ag/CHI-G hydrogels with 5 mM (**b**), 7 mM (**c**), and 11 mM (**d**) AgNO_3_.

The dense and rigid structures of the Ag/CHI-G hydrogels were captured by the SEM images. SEM images of the lyophilized hydrogels were obtained for morphological analysis of the Ag/CHI-G hydrogels (Figure 6). Figure 6a presents the SEM image of the CHI-G without addition of AgNO_3_. The densified structures of Ag/CHI-G hydrogels with the reduction in porous structures as a function of AgNO_3_ concentrations (5, 7, and 11 mM) were observed (Figure 6b–d). As reported previously, the incorporation of Ag NPs into polymeric networks can affect the porous structure, resulting in dense hydrogels [62].

### 3.4. Tissue Adhesiveness of Ag NPs-Containing CHI-G Hydrogels

**Figure 7:**
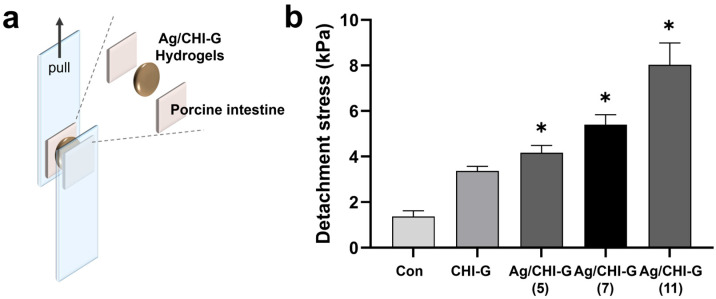
(**a**) Schematic illustration of tissue adhesiveness measurements. (**b**) Detachment stress of Ag/CHI-G hydrogels as a function of AgNO_3_ concentrations (* *p* < 0.01).

The adhesive properties of the Ag/CHI-G hydrogels were evaluated using a modified lap-shear test. Figure 7a presents the experimental procedures for the tissue adhesiveness measurements of the Ag/CHI-G hydrogels. After attaching the porcine intestinal tissues to the PET films, the Ag/CHI-G hydrogels were applied between the tissues. The tensile strength was monitored by pulling the PET films. The detachment stress of CHI-G hydrogels without AgNO_3_ was measured to be 3.4 ± 0.2 kPa, whereas Ag/CHI-G hydrogels prepared with AgNO_3_ solution (5, 7, and 11 mM) exhibited detachment stress of 4.2 ± 0.3, 5.4 ± 0.4, and 8.0 ± 0.9 kPa, respectively (Figure 7b). The fibrin glues show the 2.8 kPa of tissue adhesion strength using porcine skin tissues, as previously reported [63]. The tissue adhesiveness of Ag/CHI-G hydrogels was comparable with that of fibrin-based commercial glues. The detachment stress gradually increased as a function of the AgNO_3_ concentration, resulting in a concentration-dependent enhancement of the adhesive properties. As previously reported, the incorporation of Ag NPs into polymeric networks can significantly enhance the tissue adhesion properties of hydrogels [64]. This suggests that the Ag NPs in the CHI-G network may contribute to the reinforcement of the hydrogel network with tissue adhesiveness.

### 3.5. Cell Viability of Ag/CHI-G Hydrogels

**Figure 8:**
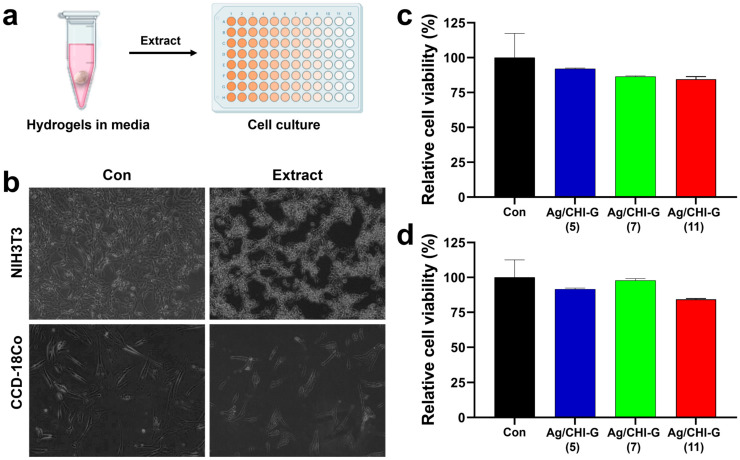
(**a**) Schematic of cell viability tests using extracts of Ag/CHI-G hydrogels. (**b**) Phase-contrast images of NIH/3T3 (top) and CCD-18Co (bottom) cells after a 24 h exposure to Ag/CHI-G hydrogel extracts. (**c**,**d**) Relative cell viability (RCV) of (**c**) NIH/3T3 and (**d**) CCD-18Co cells (Con: black, Ag/CHI-G (5): blue, Ag/CHI-G (7): green, and Ag/CHI-G (11): red).

The cytocompatibility of Ag/CHI-G hydrogels was evaluated using hydrogel extracts. Figure 8a shows the procedure for the cell viability tests of the Ag/CHI-G hydrogels. The hydrogels were immersed in the culture medium for 24 h, and the extracts were obtained and applied to the cells. Phase-contrast microscopy images (Figure 8b) show that both NIH/3T3 and CCD-18Co cells in the control and Ag/CHI-G hydrogel groups were uniformly distributed and maintained their typical fibroblast-like morphologies. The relative cell viability (RCV) of NIH/3T3 cells treated with Ag/CHI-G hydrogel extracts were 92.1 ± 0.5% at 5 mM AgNO_3_, 86.4 ± 0.5% at 7 mM AgNO_3_, and 84.5 ± 1.9% at 11 mM AgNO_3_ when the RCV values were normalized to the control (100%) (Figure 8c). For CCD-18Co cells, the RCV values were 91.6 ± 0.7% at 5 mM AgNO_3_, 97.8 ± 1.2% at 7 mM AgNO_3_, and 84.3 ± 0.5% at 11 mM AgNO_3_ (Figure 8d). Although the RCV values were slightly lower as a function of AgNO_3_ concentration, more than 80% viability was retained in all hydrogel extracts. Additionally, no significant differences (*p* > 0.5) were observed among the groups. The Ag/CHI-G hydrogels showed no significant toxicity at concentrations of 4 wt% CHI-G with 5, 7, or 11 mM AgNO_3_.

**Figure 9:**
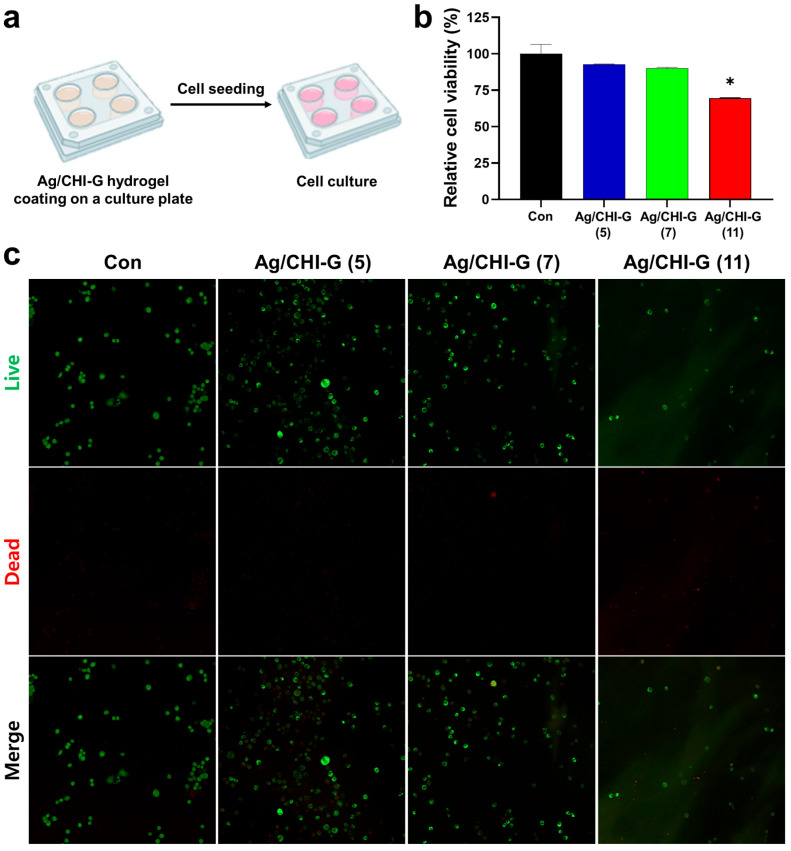
(**a**) Illustrations of procedures of live and dead tests on the Ag/CHI-G hydrogel-coated surfaces. (**b**) RCV values of NIH/3T3 cells on the Ag/CHI-G hydrogels films by MTT assay after 24 h (Con: black, Ag/CHI-G (5): blue, Ag/CHI-G (7): green, and Ag/CHI-G (11): red) (* *p* < 0.01). (**c**) Representative fluorescent (live, dead, and merge) images of NIH/3T3 cells cultured on the Ag/CHI-G hydrogels films (green: live cells and red: dead cells).

The cytocompatibility of the Ag/CHI-G hydrogels was further evaluated by direct cell culture on the Ag/CHI-G hydrogel films. As shown in Figure 9a, the hydrogels were coated onto cover glasses on the culture plates. The RCV of NIH/3T3 cells on the hydrogel films was 92.8 ± 0.2% at 5 mM AgNO_3_, 90.0 ± 0.8% at 7 mM AgNO_3_, and 69.6 ± 0.3% at 11 mM AgNO_3_ (Figure 9b). Notably, the Ag/CHI-G hydrogel films with 11 mM AgNO_3_ exhibited a reduction in cell viability, indicating that relatively high concentrations of AgNO_3_ in the hydrogels affected cytotoxicity under the direct contact conditions of cells and hydrogels. In addition, the green (live) and red (dead) cells in the fluorescence images supported the biocompatibility of the Ag/CHI-G hydrogel films (Figure 9c). Most cells displayed green fluorescence (calcein-AM, live cells) and were evenly distributed across the surface of the control and Ag/CHI-G hydrogel films with 5 and 7 mM AgNO_3_. Both quantitative analysis from the MTT assay and fluorescence images from the live/dead assay supported the cytocompatibility of Ag/CHI-G hydrogel films. However, the Ag/CHI-G hydrogel films with 11 mM AgNO_3_ showed an overall reduction in cell density, with an increase in the number of dead cells. These results suggest that optimization of concentrations of AgNO_3_ are significant for the application of Ag/CHI-G hydrogels for antimicrobial applications.

### 3.6. Antibacterial Activity of Ag/CHI-G Hydrogels

**Figure 10:**
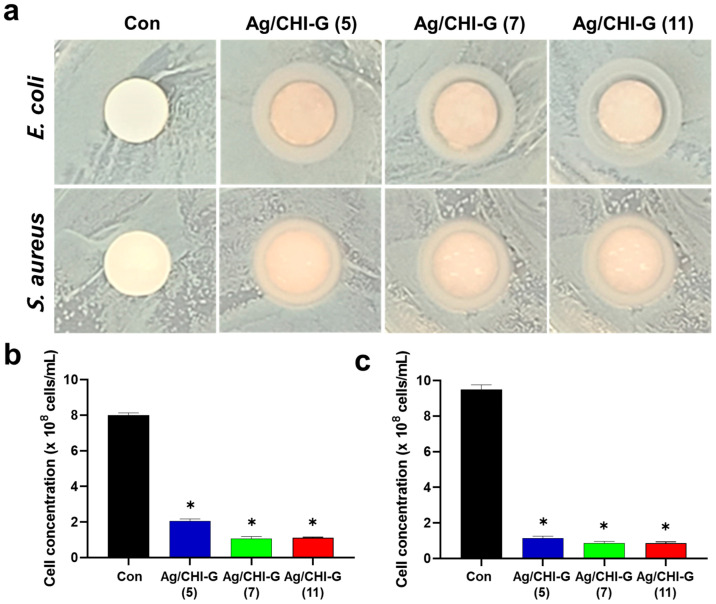
(**a**) Photographic images of zone of inhibition tests using Ag/CHI-G hydrogels against *E. coli* and *S. aureus*. (**b**,**c**) Number of (**b**) *E. coli* and (**c**) *S. aureus* in LB media with Ag/CHI-G hydrogels-applied disks (Con: black, Ag/CHI-G (5): blue, Ag/CHI-G (7): green, and Ag/CHI-G (11): red) (* *p* < 0.01).

The Ag/CHI-G hydrogels exhibited antibacterial activity against both *E. coli* and *S. aureus*. ZOI tests were performed to evaluate the antibacterial activity of the Ag/CHI-G hydrogels. As shown in Figure 10a, bacterial growth suppression was clearly observed between control and Ag/CHI-G hydrogels-applied groups with 5, 7, or 11 mM AgNO_3_ against *E. coli* and *S. aureus*. The ZOI of the Ag/CHI-G hydrogel-treated groups were significantly larger than those of the control group (Table 1). Quantitative analysis of the antibacterial activity of the Ag/CHI-G hydrogels was conducted by incubating the Ag/CHI-G hydrogels on a disk in LB medium containing both *E. coli* and *S. aureus* at an initial cell density of 2 × 10^6^ cells/mL. As shown in Figure 10b, the number of bacterial cells of *E. coli* was reduced to 2.0 × 10^8^ cells/mL for Ag/CHI-G hydrogels with 5 mM AgNO_3_ and to 1.0 × 10^8^ cells/mL for Ag/CHI-G hydrogels with 7 and 11 mM AgNO_3_, which are far lower than that of control (8.0 × 10^8^ cells/mL). Furthermore, the number of bacterial cells of *S. aureus* in the control group reached 9.5 × 10^8^ cells/mL, whereas the Ag/CHI-G hydrogel groups reduced the number of bacteria to 1.1 × 10^8^ cells/mL (5 mM AgNO_3_) and 0.87 × 10^8^ cells/mL (7 and 11 mM AgNO_3_) (Figure 10c). The Ag NPs show excellent antibacterial properties by bacterial cell membrane disruption and reactive oxygen species generation, as previously reported [65,66,67]. In addition, the Ag NPs exhibit low bacterial resistance compared to the conventional antibiotics [66,67].

Our study was limited experimental conditions. Further study is warranted to increase the antibacterial properties and reduce the biocompatibility at the high concentrations of Ag NPs in the Ag/CHI-G hydrogels. In addition, this study lacks efficacy and safety assessments of Ag/CHI-G hydrogels in vivo. Further studies are required to validate the biological relevance of the Ag/CHI-G hydrogels for treatments of wounds both in vitro and in vivo.

## 4. Conclusions

In summary, antibacterial and tissue adhesive silver nanoparticle (Ag NPs)-containing gallic acid-conjugated chitosan (CHI-G) hydrogels were prepared in this study. Ag NPs were simultaneously formed by the addition of AgNO_3_ to the CHI-G solution without additional reductants, resulting in the formation of Ag/CHI-G hydrogels. The hydrogels exhibited controllable elastic moduli depending on the concentrations of CHI-G and AgNO_3_. In addition, reduced swelling ratios, increased in vitro stability, and decreased porous structures of the Ag/CHI-G hydrogels were observed as a function of AgNO_3_ concentration. The hydrogels showed improved tissue adhesive properties compared to the CHI-G hydrogels alone. Furthermore, the hydrogels were biocompatible and exhibited antibacterial properties against *Escherichia coli* and *Staphylococcus aureus*. These findings suggest that the Ag/CHI-G hydrogels can be used as antibacterial, adhesive, and biocompatible hydrogels for wound dressings and surface coatings.

## Figures and Tables

**Table 1:** Diameter of zones of inhibition (ZOI) for Ag/CHI-G hydrogels.

Samples	Diameter of ZOI (mm)	
	*E. coli*	*S. aureus*
Ag/CHI-G (5)	11.32 ± 0.06	10.80 ± 0.02
Ag/CHI-G (7)	11.70 ± 0.12	11.06 ± 0.12
Ag/CHI-G (11)	12.46 ± 0.16	11.18 ± 0.10

## Data Availability

The data presented in this study are available on request from the corresponding author.

## Abbreviations

The following abbreviations are used in this manuscript:

CHI-G	Gallic acid-conjugated chitosan
Ag NPs	Silver nanoparticles
Ag/CHI-G	Ag NP-containing CHI-G
EDC	1-Ethyl-3-(3-dimethylaminopropyl)carbodiimide
NHS	N-Hydroxysuccinimide
DDW	Distilled and deionized water
NMR	Nuclear magnetic resonance
UV-Vis	Ultraviolet-visible
SEM	Scanning electron microscopy
G′	Elastic modulus
G″	Viscous modulus
ZOI	Zone of inhibition
RCV	Relative cell viability
